# Artificial intelligence in breast cancer survival prediction: a comprehensive systematic review and meta-analysis

**DOI:** 10.3389/fonc.2024.1420328

**Published:** 2025-01-07

**Authors:** Zohreh Javanmard, Saba Zarean Shahraki, Kosar Safari, Abbas Omidi, Sadaf Raoufi, Mahsa Rajabi, Mohammad Esmaeil Akbari, Mehrad Aria

**Affiliations:** ^1^ Department of Health Information Management, School of Allied Medical Sciences, Tehran University of Medical Sciences, Tehran, Iran; ^2^ Department of Health Information Technology and Management, School of Allied Medical Sciences, Shahid Beheshti University of Medical Sciences, Tehran, Iran; ^3^ Department of Aerospace Engineering, Khaje Nasir Toosi University of Technology, Tehran, Iran; ^4^ Department of Electrical and Software Engineering, University of Calgary, Calgary, AB, Canada; ^5^ Department of Computer Science, University of Arizona, Tucson, AZ, United States; ^6^ Department of Electrical Engineering, University of Guilan, Rasht, Iran; ^7^ Cancer Research Center, Shahid Beheshti University of Medical Sciences, Tehran, Iran

**Keywords:** breast cancer, survival prediction, machine learning, deep learning, clinical data, systematic review, meta-analysis

## Abstract

**Background:**

Breast cancer (BC), as a leading cause of cancer mortality in women, demands robust prediction models for early diagnosis and personalized treatment. Artificial Intelligence (AI) and Machine Learning (ML) algorithms offer promising solutions for automated survival prediction, driving this study’s systematic review and meta-analysis.

**Methods:**

Three online databases (Web of Science, PubMed, and Scopus) were comprehensively searched (January 2016-August 2023) using key terms (“Breast Cancer”, “Survival Prediction”, and “Machine Learning”) and their synonyms. Original articles applying ML algorithms for BC survival prediction using clinical data were included. The quality of studies was assessed via the Qiao Quality Assessment tool.

**Results:**

Amongst 140 identified articles, 32 met the eligibility criteria. Analyzed ML methods achieved a mean validation accuracy of 89.73%. Hybrid models, combining traditional and modern ML techniques, were mostly considered to predict survival rates (40.62%). Supervised learning was the dominant ML paradigm (75%). Common ML methodologies included pre-processing, feature extraction, dimensionality reduction, and classification. Deep Learning (DL), particularly Convolutional Neural Networks (CNNs), emerged as the preferred modern algorithm within these methodologies. Notably, 81.25% of studies relied on internal validation, primarily using K-fold cross-validation and train/test split strategies.

**Conclusion:**

The findings underscore the significant potential of AI-based algorithms in enhancing the accuracy of BC survival predictions. However, to ensure the robustness and generalizability of these predictive models, future research should emphasize the importance of rigorous external validation. Such endeavors will not only validate the efficacy of these models across diverse populations but also pave the way for their integration into clinical practice, ultimately contributing to personalized patient care and improved survival outcomes.

**Systematic Review Registration:**

https://www.crd.york.ac.uk/prospero/, identifier CRD42024513350.

## 1 Introduction

Breast cancer (BC) remains the most prevalent cancer and the leading cause of cancer-related mortality in women globally (1). This significant heterogeneity translates to substantial variations in individual patient survival following a BC diagnosis (2). Several factors influence BC prognosis, including patient demographics (e.g., age) (3, 4), tumor characteristics (e.g., size and lymph node involvement) (5), and tumor-derived biomarkers (e.g., hormone receptor status) (6, 7). Accurate survival prediction is crucial for understanding patient outcomes, guiding clinical decision-making, evaluating treatment efficacy, identifying prognostic factors, and developing personalized therapeutic strategies.

Artificial intelligence (AI), particularly machine learning (ML), has a pivotal role in data analysis, including medical data (8–11). These techniques offer promising avenues for enhancing BC survival prediction accuracy (12, 13). Survival analysis aims to model the time-to-event relationship, linking patient outcomes with associated variables (14, 15).

Researchers have developed various ML-based survival models to predict BC outcomes. Studies like (16) employed diverse ML classifiers, including multilayer perceptron (MLP), random forest (RF), decision tree (DT), and Support vector machine (SVM) to predict BC survival in a dataset of 4,902 patients. Additionally (17), explored deep learning (DL) approaches for predicting BC patient survival post-operatively.

Several literature reviews have emerged, discussing, and comparing outcomes of ML-derived prediction models in BC (18–22, 96). Some specifically focus on BC survival prediction, with (22) reviewing ML applications in predicting 5-year BC survival rates. Others, like (15), surveyed various ML and DL algorithms used in BC patient survival prediction. However, the rapid evolution of AI necessitates frequent review updates. Moreover, we are motivated to carry out this systematic review to synthesize and classify all AI algorithms used in BC survival prediction and present them in a structured form to help researchers select appropriate methodologies in upcoming research.

This systematic review aims to comprehensively explore the application of AI techniques in predicting BC survivability and examine the challenges associated with developing and refining these models. We will analyze all prediction models published from 2017 to 2023, evaluating their performance across diverse contexts. Through a broad analysis, we intend to investigate the advancements, challenges, and potential of AI-based BC survival prediction. Our review will encompass the spectrum of AI methods, data sources, and testing methodologies employed in prior research. Furthermore, we will critically evaluate the strengths and limitations of existing studies, with a particular focus on external validation and real-world performance assessment. By synthesizing these findings, we seek to illuminate the effectiveness, reliability, and clinical utility of ML-derived BC survival prediction models.

The remaining sections are structured as follows: Section 2 details the employed methodology, outlining eligibility criteria, reviewed databases, search strategy, study selection, and data extraction processes. Section 3 presents the review findings, encompassing the characteristics of reviewed studies aligned with conventional ML workflow steps (database selection, pre-processing, data augmentation, segmentation, feature extraction, dimensionality reduction, classification, and performance evaluation). Section 4 delves into a discussion of our findings, highlighting existing challenges and future research directions. Finally, the concluding section summarizes the key findings and their implications for clinical practice and further research.

## 2 Materials and methods

This systematic review and meta-analysis were conducted following the Preferred Reporting Items for Systematic Reviews and Meta-Analyses (PRISMA) guidelines (23), and registered in the International Prospective Register of Systematic Reviews (PROSPERO) (24) under registration number CRD42024513350.

### 2.1 Eligibility criteria

The selection of studies for this systematic review and meta-analysis was based on specific inclusion and exclusion criteria outlined in **Table 1**.

**Table 1:** Inclusion and exclusion criteria.

Inclusion Criteria	Exclusion Criteria
• Original research published as journal articles • Articles written in the English language • Full-text available papers • Studies that employed ML algorithms in BC survival prediction	• Other paper types, including conference papers, protocols, letters, book chapters, observational studies, pilot studies, reviews, and meta-analyses • Research focused on the application of ML algorithms in the field of BC, except survival prediction • Studies employed survival prediction algorithms for other diseases

### 2.2 Information sources and search strategy

A systematic search for relevant studies was conducted across three electronic databases: Web of Science, PubMed, and Scopus, encompassing the period from January 2016 to August 2023. The search strategy, developed collaboratively by two authors (M.A. and ME.A.), incorporated three core concepts: “Breast Cancer,” “Survival Prediction,” and “Machine Learning,” along with their synonyms derived from MeSH terms and standardized vocabulary. English-language journal articles were exclusively included. Details of the search strategy are provided in **Appendix A**.

### 2.3 Study selection

We employed EndNote software to manage and deduplicate the retrieved articles. Titles and abstracts were then screened independently by four reviewers (Z.J., S.Z., A.O., and S.R.) to assess eligibility based on predefined criteria. Disagreements were resolved by the other two reviewers (K.S. and M.R.). Full texts of potentially eligible studies were subsequently assessed for final inclusion.

#### 2.3.1 Quality assessment

The methodological quality of eligible studies was evaluated using the Qiao Quality Assessment tool (25). This tool encompasses five key domains: unmet need, reproducibility, robustness, generalizability, and clinical significance, further delineated by nine specific items. Studies were assessed independently by reviewers using a binary response format (‘Yes’ or ‘No’) for each item in a pre-defined table. Scores were assigned (‘1’ for ‘Yes’, ‘0’ for ‘No’), and the sum for each study determined its overall methodological quality. A pre-established threshold of 5 or more points (detailed in **Appendix B**) categorized studies as high-quality for further inclusion in the meta-analysis.

### 2.4 Data extraction

Following quality assessment, data were independently extracted from all high-quality studies using a pre-defined data extraction form created in Google Sheets. This form captured key characteristics of the included studies, including publication year, data availability, modality, number of datasets/samples, training data details (attributes, features, pre-processing, augmentation), feature selection and classification methods, model presentation, ML generation, paradigm and algorithms, time window, validation strategy, method and metrics, and best-achieved validation accuracy. Disagreements arising during extraction were resolved through discussion among all researchers.

### 2.5 Meta-analysis

In this systematic review, we extracted the best accuracy of ML models presented in studies. Then, we averaged the reported accuracies of each particular methodology to gain a holistic insight. This synchronization approach was carried out to identify the overall performance of methodologies and ease the comparison of different categories in various conditions.

## 3 Results

### 3.1 Search results

**Figure 1:**
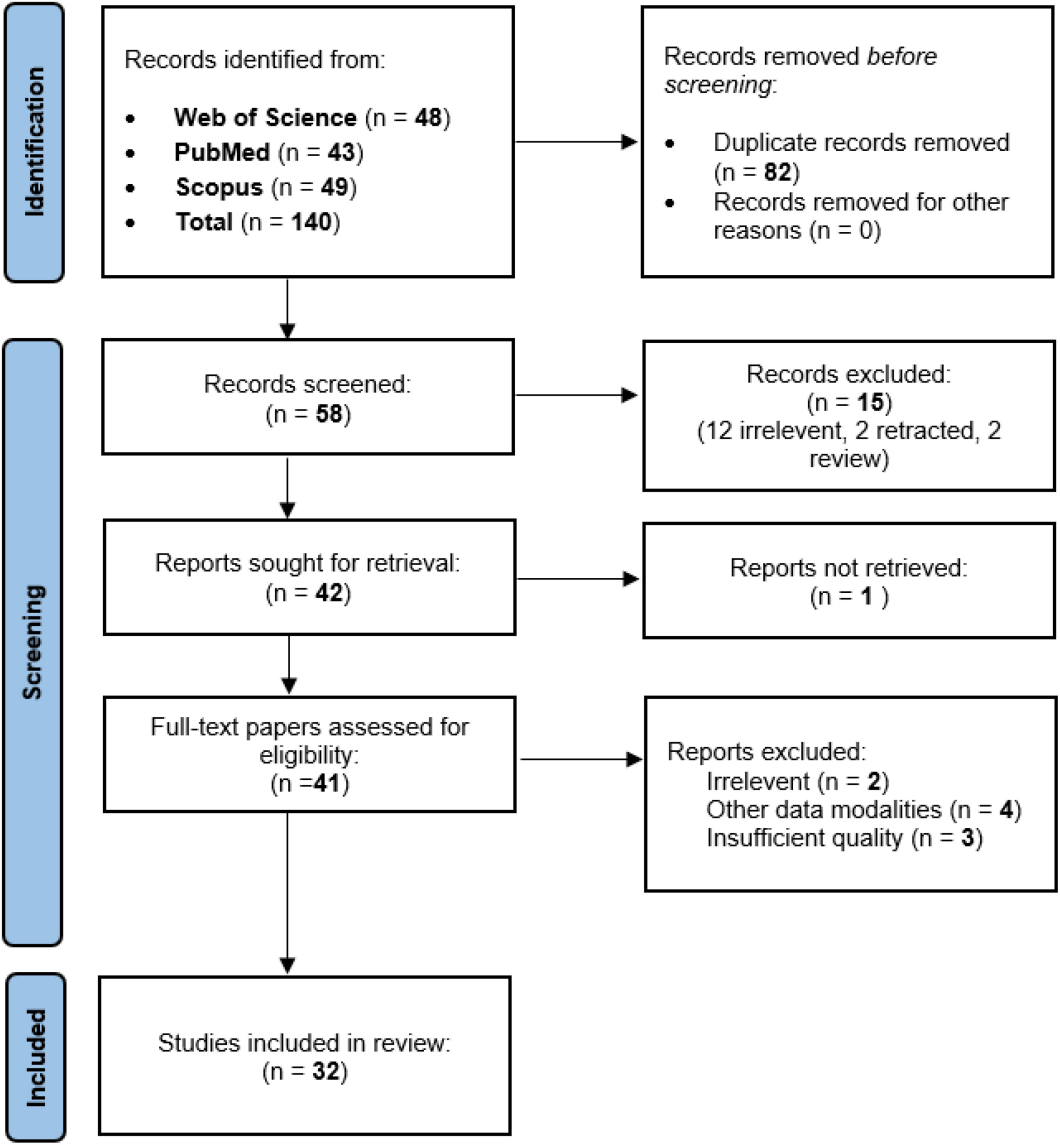
PRISMA flow diagram for searching resources.

A systematic search of three databases yielded 140 articles. After deduplication (n=82), 58 studies underwent initial screening. Ultimately, 32 articles met the pre-defined inclusion criteria (see PRISMA diagram, **Figure 1**).

### 3.2 Data extraction

Data from the 32 included studies were systematically extracted using a pre-defined form. Extracted characteristics are presented in tabular format within **Appendix C**.

### 3.3 Characteristics of the included studies

As shown in **Table 2**, a marked increase in studies utilizing ML algorithms for BC survival prediction and classification is evident. The majority of included studies (n=26, 81.3%) were published between 2019 and 2023.

**Table 2:** Chronological distribution of the selected studies.

Year	No. Contributions	In terms of %	Accuracy (%)
2016	1	3.12	87.90
2018	3	9.37	88.86
2019	5	15.63	90.38
2020	1	3.13	72
2021	7	21.88	94.04
2022	8	25	91.25
2023	7	21.88	87.28
	32		89.73

### 3.4 Machine learning methods

#### 3.4.1 ML generation

Established machine learning methods categorized as “Traditional”, prioritize well-understood algorithms with strong theoretical underpinnings and interpretable results (e.g., K-nearest neighbors). “Modern” approaches leverage deep neural networks inspired by the brain’s structure and function (e.g., convolutional neural networks), alongside nature-inspired metaheuristic algorithms. “Hybrid” techniques strategically combine these approaches to capitalize on their complementary strengths. The integration of ML and DL applications in medical decision-making has witnessed significant growth in recent years, often complementing traditional methods (26–30). Modern ML leverages automated, integrated algorithms, particularly multi-layer neural networks, offering a unified learning framework that addresses the limitations of traditional approaches by eliminating the need for separate feature extraction and classification stages (31). Common modern techniques include Convolutional Neural Networks (CNNs), Deep Neural Networks (DNNs), Long Short-Term Memory (LSTMs), Recurrent Neural Networks (RNNs), ensemble methods like Random Forests, and XGBoost.

Traditional ML algorithms typically rely on a sequential approach with distinct stages for feature extraction and classification (32). Support Vector Machines (SVMs), K-Means Clustering, Artificial Neural Networks (ANNs), Decision Trees (DTs), AdaBoost, and Naive Bayes (NB) are commonly employed traditional algorithms.

**Figure 2:**
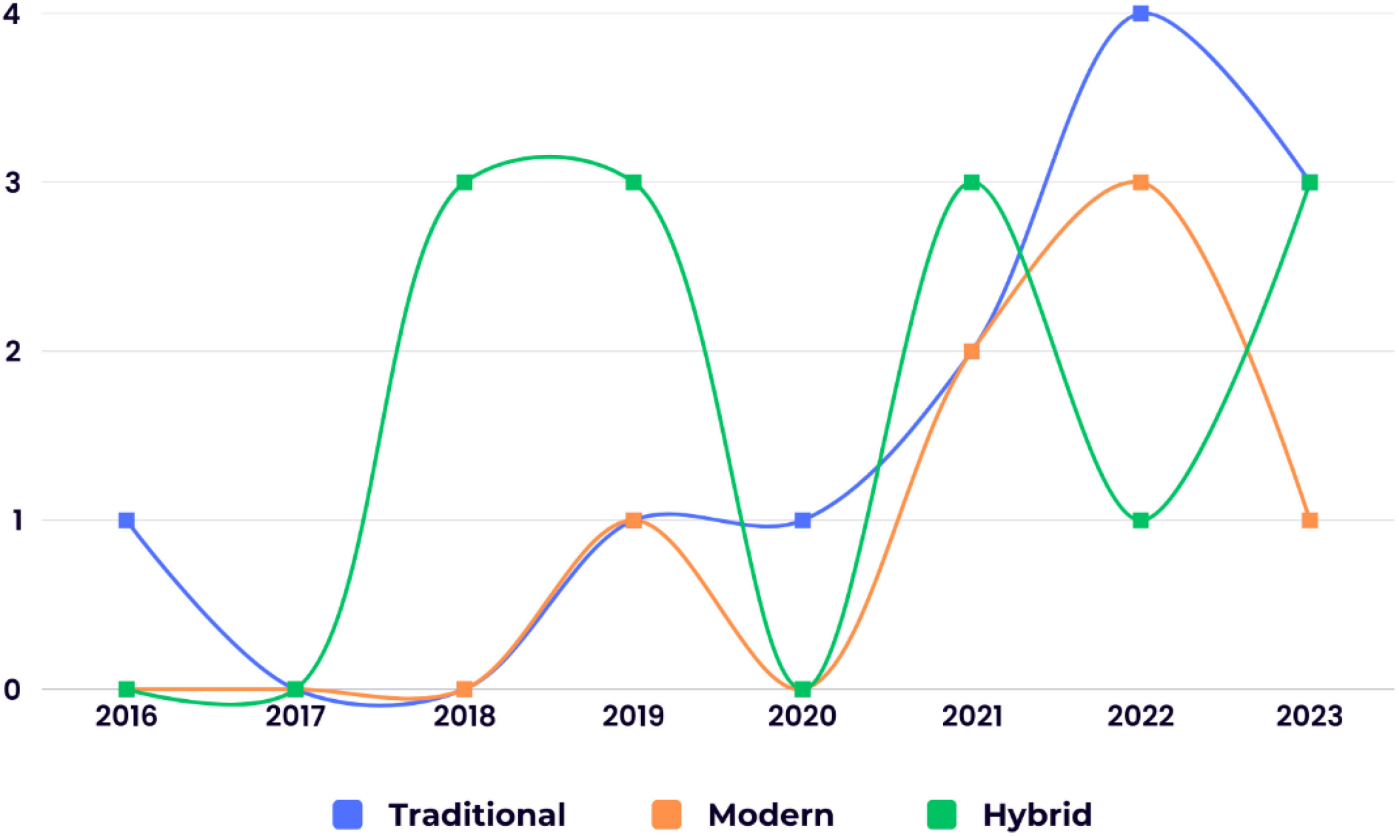
The chronological distribution of the studies utilizing different ML generations in BC survival prediction.

This review analyzed the distribution of ML algorithm generations used for BC survival prediction. Modern techniques were employed in 21.87% (7 studies) of the included studies with an average accuracy of 88.72%. Traditional techniques were used in 37.5% (12 studies) with an average accuracy of 87.23%, while hybrid methods (combining modern and traditional approaches) were used in 40.62% (13 studies) with an average accuracy of 91.73%. **Figure 2** depicts the chronological trend of ML generation usage in BC survival prediction studies across the eight years examined.

#### 3.4.2 ML paradigm

ML encompasses four primary learning paradigms: supervised, unsupervised, semi-supervised, and reinforcement learning (33). Supervised learning utilizes labeled data for training, encompassing popular algorithms like DT and SVM. Conversely, unsupervised learning trains models using unlabeled data (34, 35). Semi-supervised learning leverages both labeled and unlabeled data, while reinforcement learning focuses on adapting to an environment through trial and error (33, 36).

**Figure 3:**
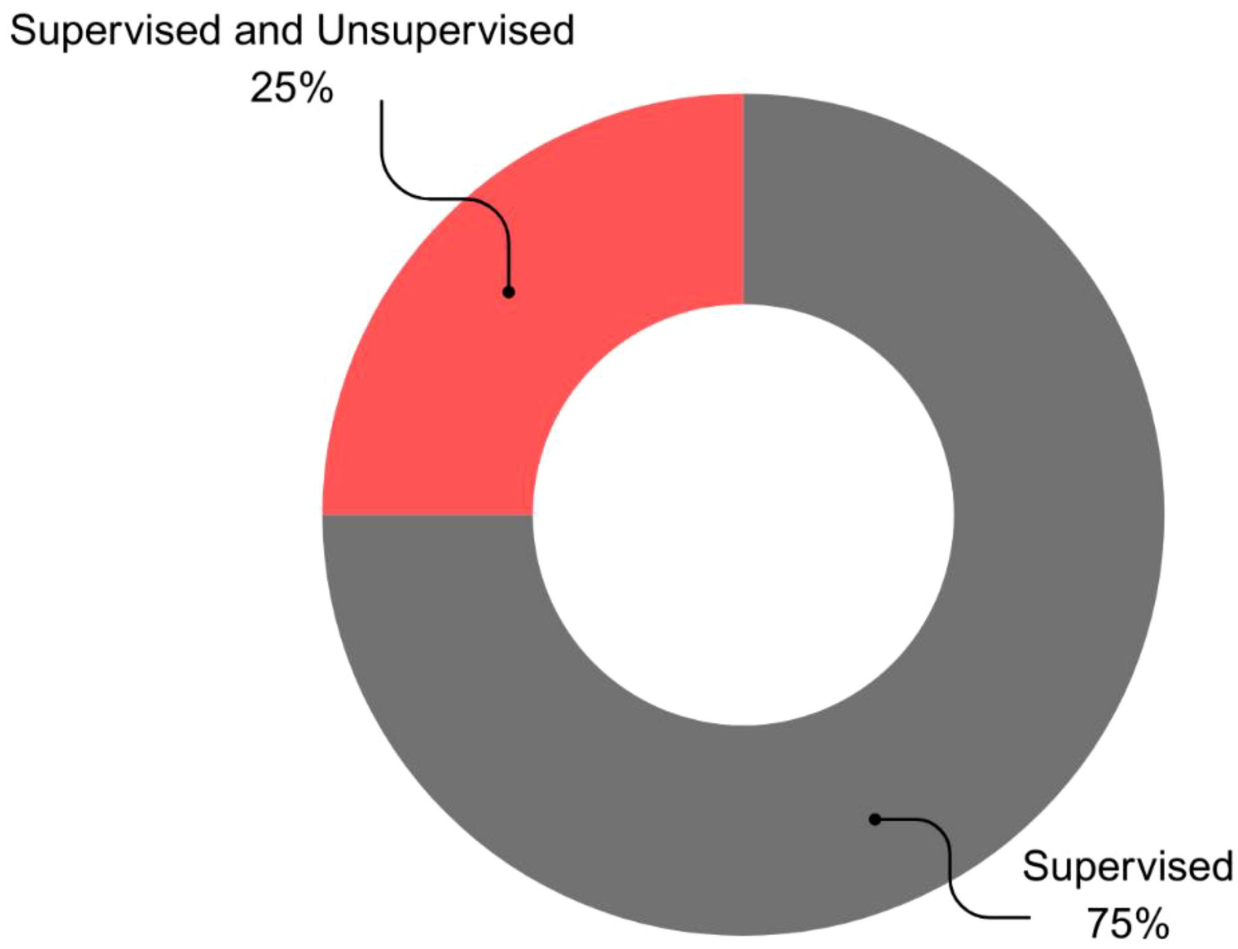
Analysis of the ML Paradigm of the proposed BC survival prediction methods.

This review examined the distribution of ML paradigms for BC survival prediction. Supervised learning emerged as the dominant paradigm, employed in 75% (n=24) of studies with an average accuracy of 88.82%. The combination of supervised and unsupervised learning was the next most frequent (25%, n=8), achieving an average accuracy of 92.07%. Notably, none of the studies utilized semi-supervised, reinforcement, or unsupervised learning alone (**Figure 3**).

### 3.5 Datasets

The reviewed studies employed a variety of BC datasets.

#### 3.5.1 Data availability and analysis

Private datasets were the predominant choice, utilized in over half of the studies (56.25%, n=18, acc=89.42%). Notably, the use of private datasets has increased since 2022. Publicly available datasets, including established resources like The Cancer Genome Atlas (TCGA) (37), Molecular Taxonomy of Breast Cancer International Consortium (METABRIC) (38), and Surveillance, Epidemiology, and End Results (SEER) (39), were used in a smaller proportion of studies (34.37%, n=11, acc=89.80%). The characteristics of publicly available datasets are detailed in **Table 3**. A small subset of studies (9.37%, n=3, acc=93.1%) employed a combination of both public and private data.

**Table 3:** Publicly available datasets used for BC survival prediction.

Dataset	Year	Source	Year of Studypublication	No. Samples	No. Patients	Data Modality
**METABRIC** (38)	2012	European Bioinformatics Institute	2018-2019-2020-2022	2000	2000	Clinical + Omics
**SEER** (39)	1975-2020	National Cancer Institute of the USA	2018-2021-2022	4,917,840	4,917,840	Clinical
**Wisconsin breast cancer (WBC)** (40)	1992	UCI Machine Learning Repository	2021	699	699	Image
**Wisconsin Diagnosis Breast Cancer (WDBC)** (41)	1995	UCI Machine Learning Repository	2021	569	569	Image
**Wisconsin Prognosis Breast Cancer (WPBC)** (42)	1995	UCI Machine Learning Repository	2021	198	198	Image
**Mammographic Mass Dataset (MM-Dataset)** (43)	2007	UCI Machine Learning Repository	2021	961	961	Image
**The Cancer Genome Atlas (TCGA)** (37)	2006-2023	NCI and the National Human Genome Research Institute of the USA	2018-2019-2021	–	44,451	Clinical + Omics
**The Cancer Imaging Archive (TCIA)** (44)	2013	National Cancer Institute (NCI)	2019	3,268,644	–	Clinical + Image

The number of datasets per study ranged from 1 to 4. The majority of studies (75%, n=24, acc=88.52%) relied on a single dataset. Among these, METABRIC (n=4, 12.5%, acc=89.8%), SEER (n=4, 12.5%, acc=87.58%), and TCGA (n=3, 9.38%, acc=93.1%) were the most frequently used. Other, less common datasets included WBC (40) (n=2, 6.25%, acc=98.62%), WDBC (41) (n=1, 3.13%, acc=99.04%), WPBC (42) (n=1, 3.13%, acc=99.04%), MM-Dataset (43) (n=1, 3.13%, acc=99.04%), and TCIA (44) (n=1, 3.13%, acc=81%). Furthermore, 32.11% of studies (n=7, acc=96.57%) incorporated two datasets, while one study utilized four datasets.

#### 3.5.2 Training data partitioning

The reviewed studies employed various training data partitioning strategies. The majority (93.75%, n=30, acc=89.34%) utilized a single dataset for training, potentially encompassing it for both training and testing phases. A smaller subset (6.25%, n=2, acc=99.04%) employed multiple datasets specifically for training.

#### 3.5.3 Sample size and data modality

**Figure 4:**
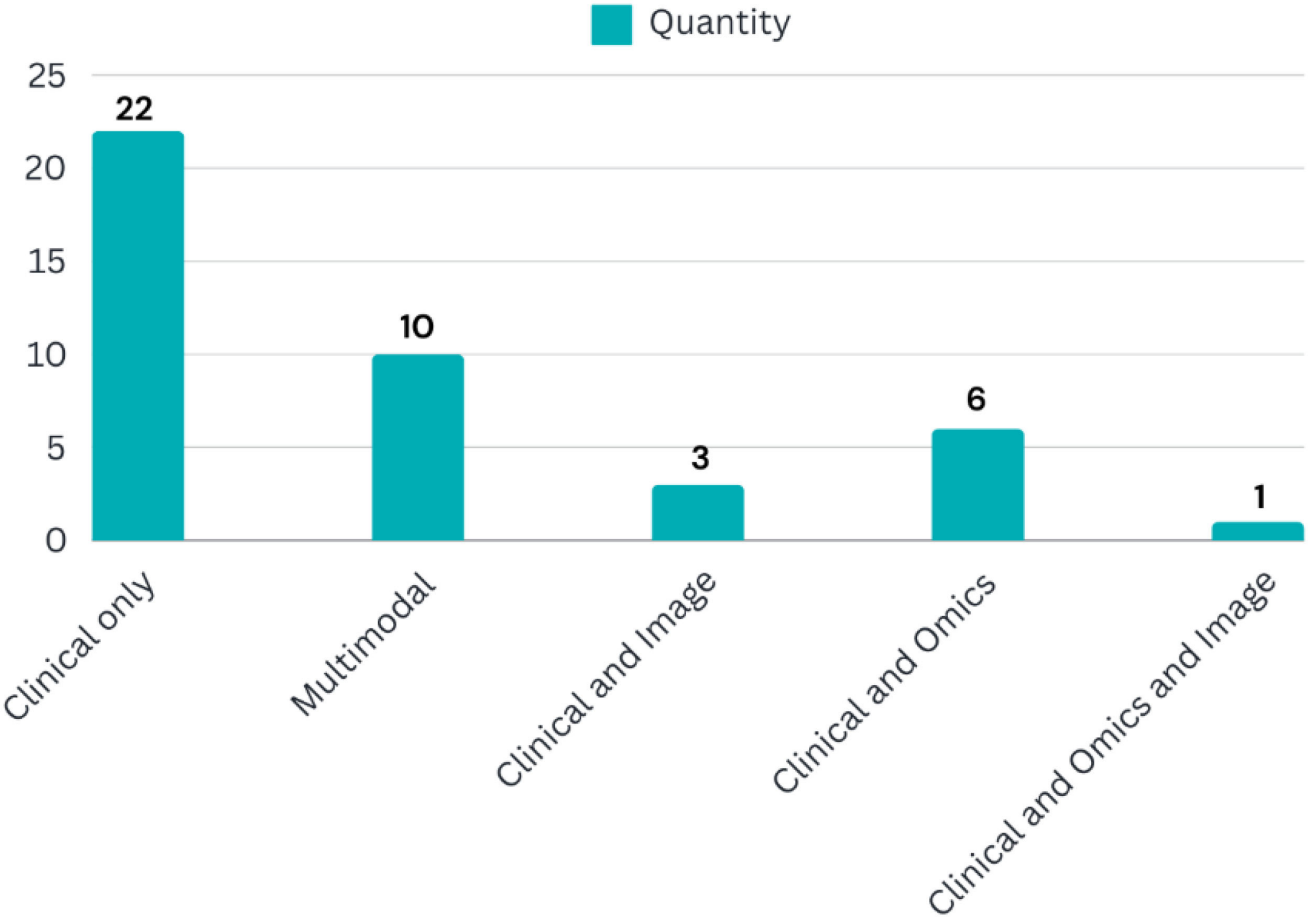
Distribution of data modalities among reviewed studies.

The datasets used in the studies exhibited significant variation in sample size. Values ranged from 38 to 163,413 cases per dataset, with an average of 12,041 cases. However, two studies lacked information regarding sample size. Additionally, the studies incorporated diverse data modalities, as illustrated in **Figure 4**.

#### 3.5.4 Time window analysis

The reviewed studies employed various time windows to evaluate prediction accuracy. The 5-year window emerged as the most frequently explored timeframe, with 12 studies (37.5%) reporting an average accuracy of 86.81%. Notably, accuracy rates appeared to peak at the 10-year window, with 5 studies (15.625%) achieving an impressive 97.95% average accuracy. Conversely, shorter windows (1 and 3 years) yielded lower accuracy rates, with only 1 study (3.125%) in each achieving accuracy above 90%. Time windows exceeding 10 years also resulted in an average accuracy of 89.23% across 5 studies (15.625%). These findings suggest a potential trend of increasing accuracy with longer prediction horizons, although further investigation is warranted.

### 3.6 Features

The studies incorporated a variety of BC features for model development. The number of features employed ranged from 5 to 625. However, four studies (12.5%, acc=94.02%) did not report the specific features utilized in their ML models.

Several studies incorporated clinicopathological features directly related to BC, including age, cancer stage, grade, tumor size, estrogen receptor (ER) status, progesterone receptor (PR) status, and human epidermal growth factor receptor 2 (HER2) status. A good description of the predictors may be accessed in (45).

### 3.7 Methodology for breast cancer survival prediction

#### 3.7.1 Pre-processing

Pre-processing is a critical step that transforms raw data into a format suitable for ML algorithms, ultimately enhancing model performance and accuracy (46, 47). Common techniques include data normalization (e.g., standardization, rescaling), augmentation, segmentation, and feature selection. Pre-processing also improves data quality by handling missing values, reducing noise (48, 49), correcting anomalies, and ensuring compatibility with modeling algorithms (50, 51).

This review found that 78.125% (n=25, acc=89.54%) of studies acknowledged employing at least one pre-processing technique. Various standardization methods were utilized, including mean removal and unit variance scaling (52), Transformation and resampling techniques (14, 16, 53, 54), data integration (16), SMOTE for imbalanced data (55), outlier detection with boxplots (56), one-hot encoding for categorical data (57), segmentation (58), and min-max scaling (59, 60) were also reported.

For missing value imputation, some studies employed methods like DL and K-Nearest Neighbors (KNN) (61), R mice package Predictive mean matching (PMM) (62), multiple imputation (MI) (56), Missing value estimation (63), or manual imputation (16, 63–67). Notably, 7 studies (21.875%, acc=90.05%) did not explicitly mention their pre-processing techniques.

##### 3.7.1.1 Data augmentation

Data augmentation is a critical pre-processing technique that mitigates the risk of overfitting in ML models by artificially expanding the training data (68–70). This is achieved through methods like generating new data samples, modifying existing data (e.g., cropping, flipping, rotating), or both (71, 72). None of the reviewed studies utilized data augmentation techniques, so the effect of incorporating this method needs further investigation.

##### 3.7.1.2 Feature selection and dimensionality reduction

**Figure 5:**
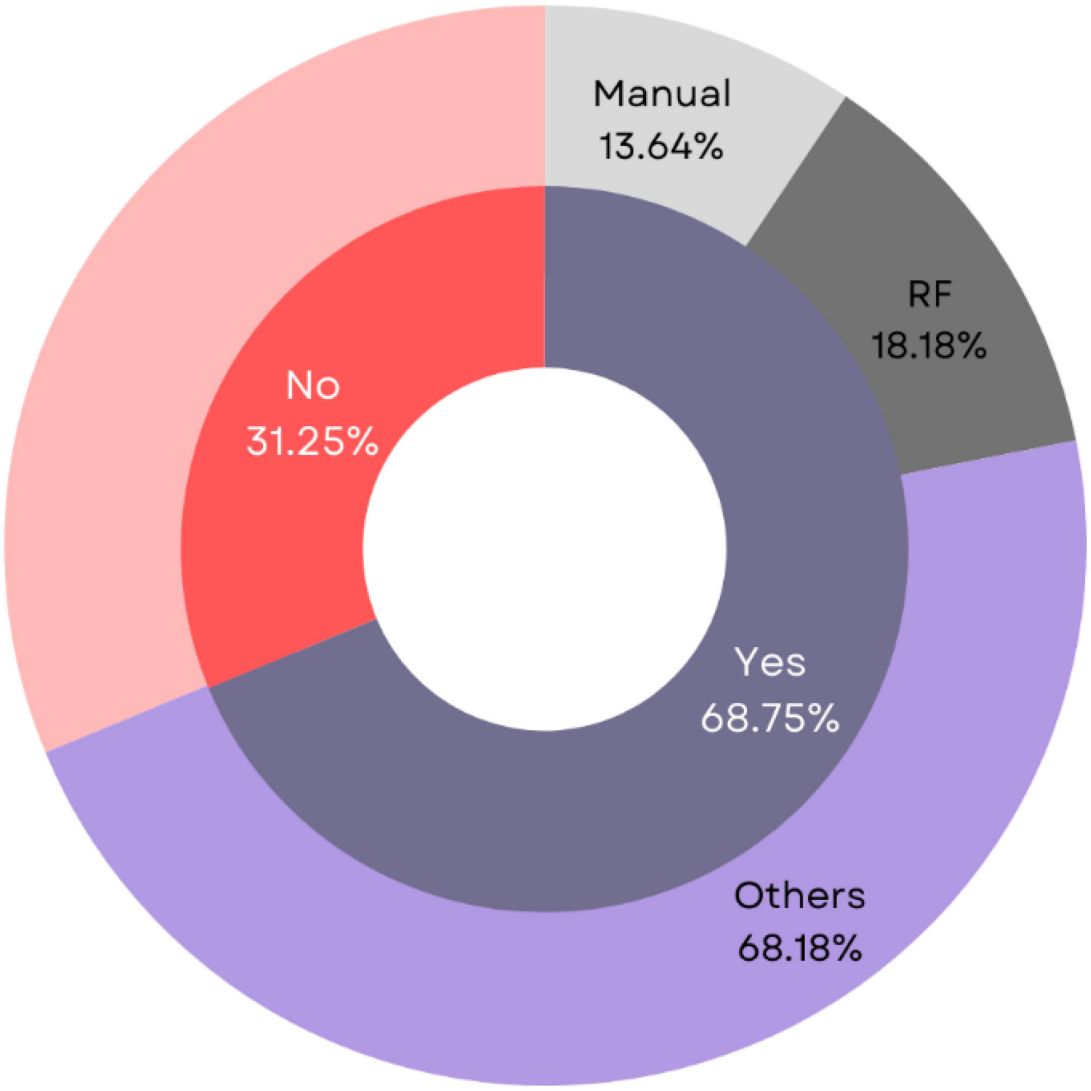
Analysis of different dimensionality reduction techniques employed for BC survival prediction.

Dimensionality reduction is an essential step that improves ML model performance by selecting relevant features and eliminating redundant ones (73, 74). Common feature selection techniques include Principal Component Analysis (PCA), Probabilistic PCA (PPCA), and Linear Discriminant Analysis (LDA). This review found that most studies (n=22, 68.75%, acc=89.08%) employed a combination of these methods, while a smaller portion (n=10, 31.25%, acc=91.11%) did not utilize any feature selection technique (**Figure 5**).

Within the studies that employed feature selection and dimensionality reduction, supervised methods were the dominant choice (46.875%, n=15, acc=87.88%). Unsupervised techniques were implemented in a smaller subset of studies (21.875%, n=7, acc=91.28%). Notably, only one study (31.25%, acc=99.04%) utilized a combination of both approaches (75).

The following sections categorize and detail the various feature selection techniques reported in the reviewed studies, differentiating between traditional and modern methods.

###### 3.7.1.2.1 Traditional feature selection techniques

This review identified traditional methods as the feature selection approach of choice in 31.25% (n=10, acc=89.24%) of the studies. Commonly employed techniques included manual selection (n=3, 9.38%, acc=89.70%), K-means clustering (n=2, 6.25%, acc=82.55%), PCA (n=2, 6.25%, acc=96.68%), minimum Redundancy Maximum Relevance (mRMR) (n=2, 6.25%, acc=94.60%), and Recursive Feature Elimination (RFE) (n=2, 6.25%, acc=99.04%).

For instance, studies like (66) employed manual selection, where researchers identified and compared features to determine the most significant ones. Similarly (17), incorporated medical expertise for feature selection.

• Supervised Traditional Feature Selection Methods

Approximately 15.63% (n=5, acc=90.13%) of the studies utilized supervised traditional techniques, including K-means clustering (76), and RFE (75, 77). Other reported methods were Variable Sensitivity Ratio (VSR) (65) and Backward Elimination (BE) (54).

• Unsupervised Traditional Feature Selection Methods

Unsupervised traditional methods were implemented in 15.63% (n=5, acc=88.58%) of the reviewed studies. These techniques included PCA (78, 79), mRMR (59, 63), and LightGBM (80).

###### 3.7.1.2.2 Modern feature selection techniques

Modern feature selection techniques, minimizing human intervention, were prevalent in the reviewed studies (n=12, 37.5%, acc=88.97%).

• Supervised Modern Feature Selection Methods

Supervised modern techniques were the dominant choice for feature selection within this category (n=10, acc=87.04%). The most frequently used methods were RF (n=4, 12.5%, acc=86.27%), CNNs (n=2, 6.25%, acc=91.92%), and SHAP (n=2, 6.25%, acc=90.00%).

• Unsupervised Modern Feature Selection Methods

Modern unsupervised techniques were employed by a smaller proportion of studies (n=2, acc=96.68%) for feature selection. PCA was the method of choice in both studies for identifying important features (53, 75).

#### 3.7.2 Classification

Classification is a fundamental objective in many healthcare-oriented ML projects (81–83). In BC survival prediction, classification algorithms aim to distinguish between patients who succumb to the disease and those who survive. Various ML techniques are employed to achieve this goal.

This review found that supervised learning was the dominant approach for classification tasks in all reviewed studies (n=32, 100%). These studies achieved an average accuracy of 89.73% in classifying BC patient survival. Notably, two studies (6.25%, avg. accuracy=94.95%) explored the use of unsupervised classification algorithms alongside supervised techniques.

The following sections categorize and detail the various classification techniques identified in the reviewed studies, differentiating between traditional and modern methods.

##### 3.7.2.1 Traditional classification techniques

Traditional classification algorithms were employed in 62.5% (n=20, acc=89.38%) of the reviewed studies. Notably, some studies implemented multiple classifiers. For example (56), used C5.0 and RIPPER to classify BC patient survival, and (66) employed both ANN and LR for BC survivability estimation.

• Supervised Traditional Classifiers

Supervised traditional methods were the dominant choice within the traditional category (n=20, 62.5%, acc=89.38%). The most frequent method was SVM (n=12, 37.5%, acc=89.24%). Other commonly used classifiers included DT (n=11, 34.37%, acc=88.74%), Neural Networks (n=10, 31.25%, acc=87.52%), NB (n=6, 18.75%, acc=96.20%), KNN (n=5, 15.625%, acc=94.51%), and AdaBoost (n=4, 12.5%, acc=93.0%).

• Unsupervised Traditional Classifiers

Only one study (3.125%, accuracy=93.1%) utilized unsupervised traditional classification. The authors employed K-means clustering for predicting survival curves in BC patients (14).

##### 3.7.2.2 Modern classification techniques

Modern classification algorithms were adopted in over 87.5% of the studies (n=28, acc=90.45%), demonstrating a notable increase since late 2018. For instance (54), developed a WTTE-RNN model to determine BC recurrence probability, and (67) used CNNs to predict BC survival time windows.

• Supervised Modern Classifiers

Supervised methods were the prevalent choice for modern classification (n=28, acc=90.45%). Deep Neural Networks (DNNs) were frequently employed (n=12, 37.5%, acc=91.14%). Among DNNs, CNNs (n=6, 50%, acc=90.57%) were most common, followed by Dense Neural Networks (n=5, 41.67%, acc=91.89%), LSTM (n=2, 16.67%, acc=98.0%), and RNN (n=1, 8.33%, acc=91.0%). Bagging algorithms (n=16, 50%, acc=90.46%) and XGBoost (n=10, 31.25%, acc=90.77%) were also widely used.

• Unsupervised Modern Classifiers

The use of modern unsupervised classification algorithms was infrequent, with only one study (3.125%, accuracy=96.8%) employing Restricted Boltzmann Machines (RBM) for BC survivability prediction (17).

### 3.8 Performance evaluation analysis

This section analyzes the performance metrics and validation methods employed in the reviewed studies.

#### 3.8.1 Evaluation metrics

Accuracy emerged as the dominant evaluation metric, utilized in 78.13% (n=25) of the studies. Consequently, it serves as the primary basis for performance comparison across the reviewed studies (details in the Validation Accuracy section).

#### 3.8.2 Validation methods

Two primary validation approaches were identified: internal and external validation. Internal validation assesses model performance using the training data, while external validation employs a separate dataset, improving generalizability (25).

**Figure 6:**
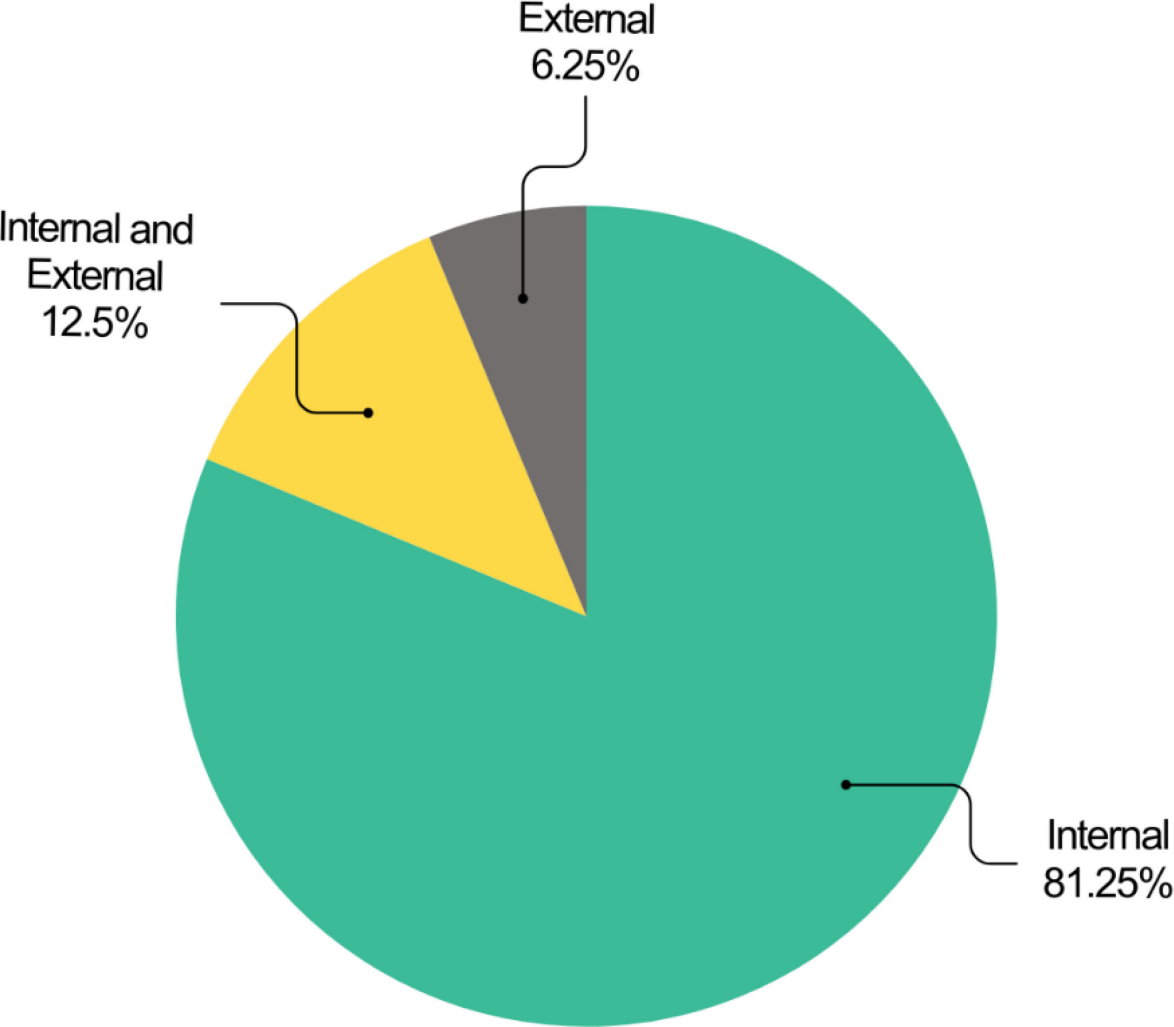
Distribution of the studies based on the used validation methods.

Analysis revealed that internal validation was the predominant approach (81.25%, n=26, acc=89.20%). A smaller portion of studies utilized external validation (6.25%, n=2, acc=89.55%), and four studies (12.5%, acc=93.4%) combined both methods (**Figure 6**).

**Table 4** details the studies that employed external validation for performance evaluation.

**Table 4:** Characteristics of the studies that utilized external validation.

Study	Year	ML Generation	ML Paradigm	Data Availability	No. Datasets	No. Samples	Pre-Processing	Feature Selector	Classifier	Evaluation Metrics	Validation Accuracy (%)
**H. Li, et al.** (62)	2016	Traditional	Supervised	Private	2	774	Handling missing data, Feature Selection	RF	RF	Accuracy, Sensitivity, Specificity, C-index	87.9
**N. Arya; S. Saha** (63)	2018	Hybrid	Both Supervised and Unsupervised	Public	2	1980	Handling missing data, Feature Selection, Data normalization	mRmR	CNN, RF	Accuracy, Sensitivity, Specificity, Precision, MCC (Matthew’s correlation coefficient)	91.2
**Q. T. N. Nguyen, et al.** (84)	2023	Hybrid	Supervised	Private	1	3914	Handling missing data, Feature Selection	SHAP	LR, LDA, LightGBM, RF, XGBoost, AdaBoost, ANN, Ensemble	Accuracy, Sensitivity, Precision, F1-score	90
**T. R. Mahesh, et al.** (55)	2021	Hybrid	Supervised	Public	2	569	Data normalization	–	NB, DT, RF, XGBoost, Reduced Error Pruning Tree	Accuracy, Time Taken to Build Model, F1 Score, Mean Absolute Error, Root Mean Squared Error, Relative Absolute Error, Root Relative Squared Error	98.2
**R. Albusayli, et al.** (85)	2021	Hybrid	Supervised	Both Private and Public	2	429	–	–	CNN, SVM	C-index	–
**L. C. Ji, et al.** (86)	2023	Hybrid	Supervised	Private	2	420	–	–	DT, RF, MLP, XGBoost, LR, NB	Accuracy	92

#### 3.8.3 Validation strategy

**Figure 7:**
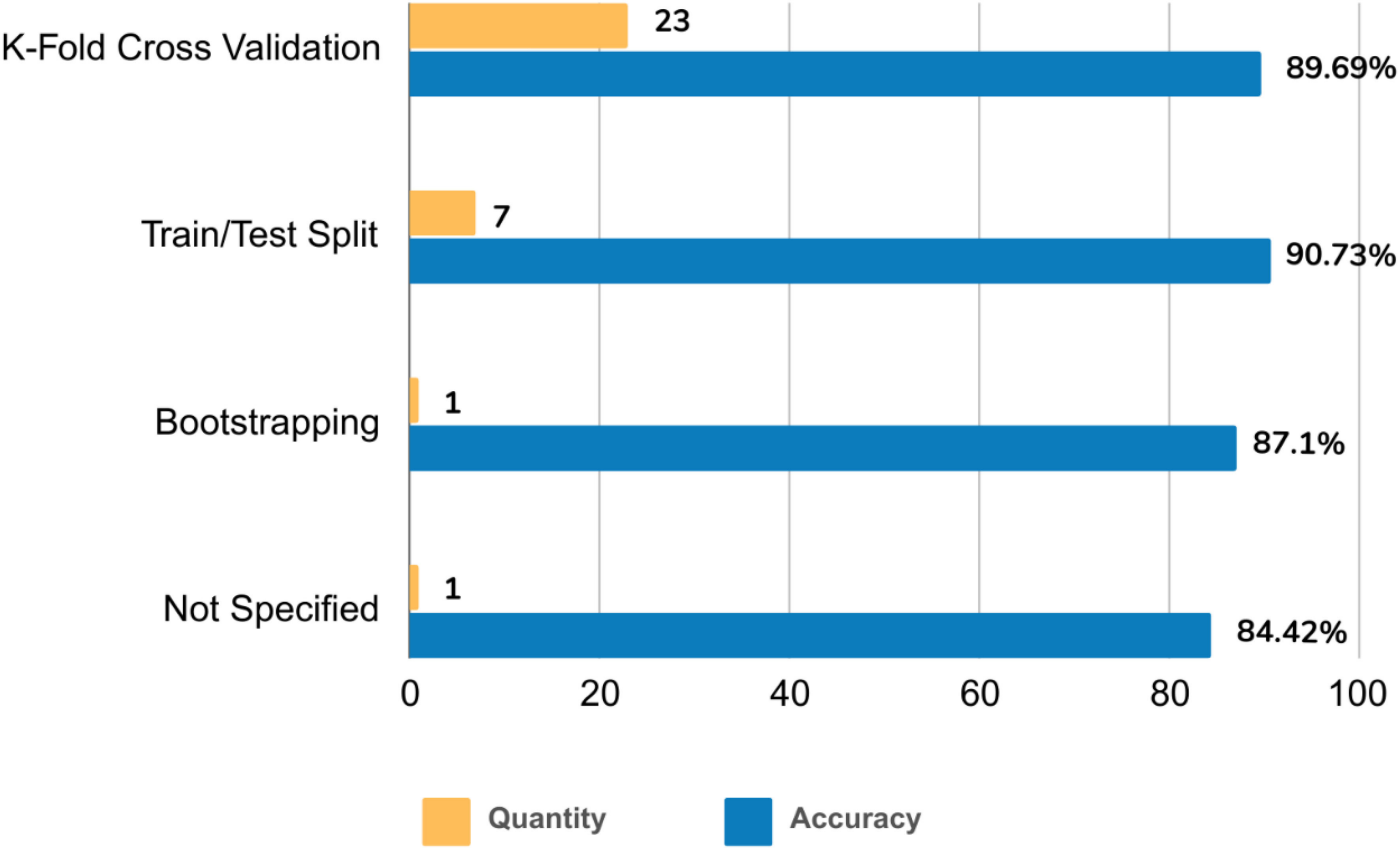
Analysis of the number and accuracy of the studies based on the used validation strategy.

The reviewed studies employed various validation strategies. K-fold cross-validation emerged as the dominant approach (71.88%, n=23, acc=89.69%). The train/test split method followed in prevalence (21.88%, n=7, acc=90.73%). Notably, bootstrapping was the least frequently used strategy (3.13%, n=1, accuracy=87.1%). One study (3.13%, accuracy=84.42%) did not report the validation strategy (**Figure 7**).

#### 3.8.4 Validation accuracy

To comprehensively assess the validation performance of the reviewed ML models, a statistical analysis of their accuracies was conducted. This analysis included minimum, maximum, average, median, variance, and standard deviation (**Table 5**).

**Table 5:** Performance of ML-based BC survival prediction methods.

Metric	Validation Accuracy
Min.	72%
Max.	99.04%
Avg.	89.73%
Median	91%
Variance	50.005
St.D.	7.07
Count	26/32

**Figure 8:**
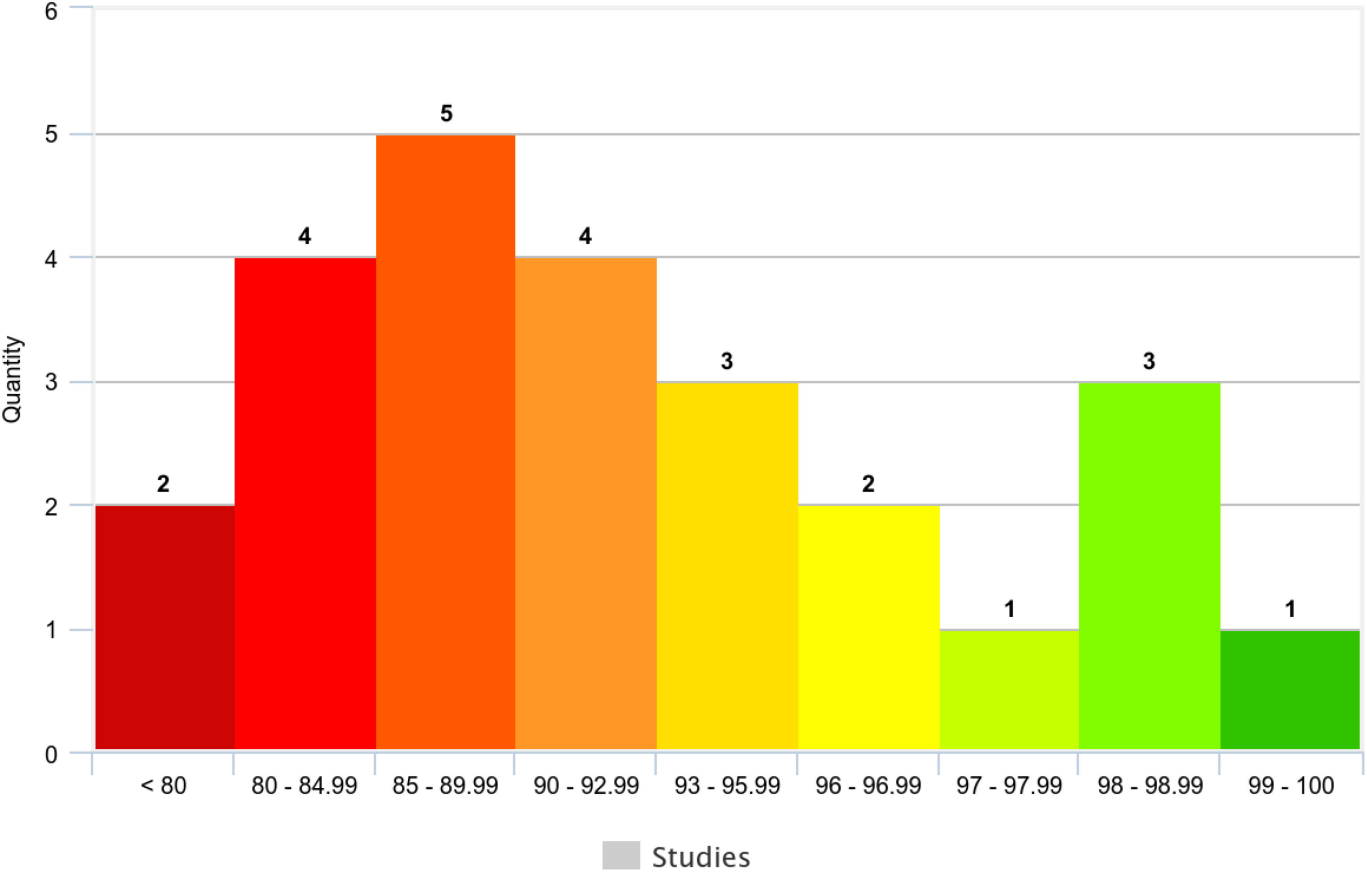
Accuracy histogram of ML-based BC survival prediction methods.

Across the 25 studies reporting validation accuracy (78.13% of the total), model accuracy ranged from 72% to 99.04% (**Table 5**). The average accuracy was 89.73%, and the median accuracy was 91% (n=32). **Figure 8** visually depicts the distribution of accuracy scores within the reviewed studies.

### 3.9 Model presentation

**Figure 9:**
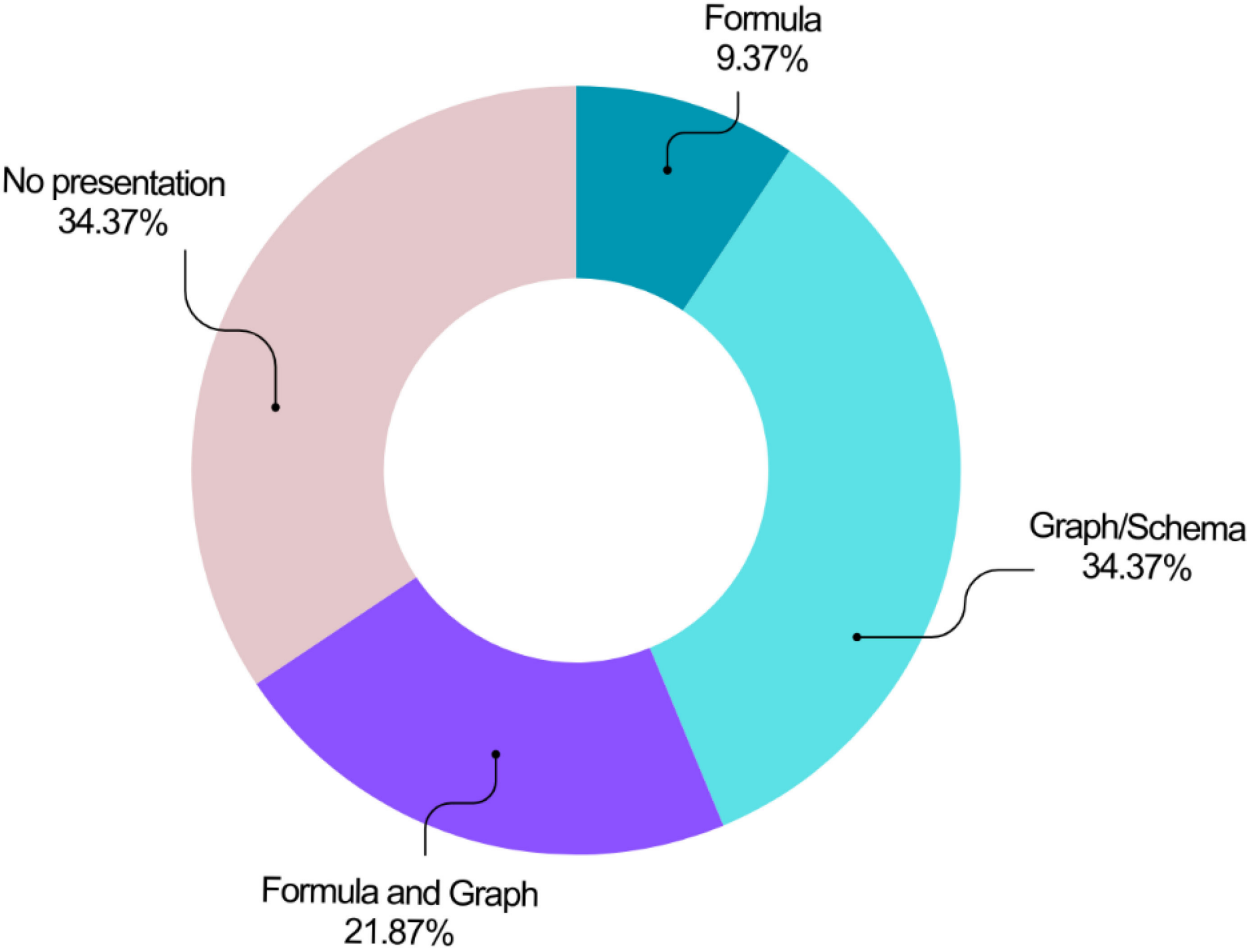
Distribution of the studies based on the model presentation approach.

The reviewed studies employed various methods to present their models (**Figure 9**). Graphs or schematics were the most prevalent approach (n=11, 34.38%), followed by a combined presentation using graphs and formulas (n=7, 21.88%). Formula-only presentations were the least common (n=3, 9.38%). Notably, over a third of the studies (n=11, 34.38%) did not visually represent their models or utilize equations.

## 4 Discussion

This systematic review and meta-analysis investigated the application of artificial intelligence in breast cancer survival prediction research published between 2016 and 2023. The analysis of 32 eligible studies revealed several noteworthy findings.

A diverse range of ML methods were employed for BC survival prediction, with an overall mean validation accuracy of 89.91%. Traditional methods remained popular, used in 25 studies (78.1%) either solely or combined with modern techniques. Studies like (20) also reported the prevalence of traditional ML models in BC recurrence prediction. However, a recent surge in modern techniques, particularly deep learning, is evident. Studies like (87, 88) highlighted the growing use of deep learning algorithms in BC detection.

Private datasets (56.25%) were more commonly used than public datasets (34.38%) for BC survival prediction. This aligns with the findings of (20). However, some studies like (22, 87) emphasized the wide use of public datasets like METABRIC, SEER, and TCGA for BC diagnosis. Notably, reviewed studies utilizing multiple public datasets achieved the highest validation accuracies (e.g., 99.04% and 98.2% in (55, 75)). This suggests the potential benefit of standard and valuable data from public sources for enhancing ML model performance.

The number of training datasets significantly impacted the results. Studies using multiple datasets (n=8) achieved a superior mean validation accuracy (95.76%). This finding emphasizes the importance of employing diverse training data to mitigate challenges associated with limited or inappropriate datasets. Furthermore, because of the emergence of different BC data modalities in recent decades and their impact on the detection and prediction of BC (89, 90), we extracted this data from datasets used in studies as well. Review of included studies showed that no study, except (85), employed all BC data modalities to predict BC survival using AI-based methodologies. This issue strengthens the necessity of utilizing diverse data to gain more precise and trustful results.

The 5-year window was the most prevalent timeframe for BC survival prediction, aligning with the established threshold for assessing survival rates as reported in (22). However, studies with a 10-year window achieved the highest mean validation accuracy (97.95%) (e.g (31, 65)). This suggests a potential benefit from considering longer prediction horizons.

The number of features used in the reviewed studies ranged from 5 to 625. Similar reviews like (19, 20, 22) reported findings on features like age, tumor size, grade, and stage as crucial predictors in BC prognostic models.

Pre-processing was a common step, with most studies employing at least one technique. Data augmentation, a valuable method for managing unbalanced or limited data, was absent in all reviewed studies. Future research should consider data augmentation to strengthen prediction models by addressing restricted data issues, as suggested in (20).

Over 68% of the studies employed feature selection techniques, primarily modern supervised methods. However, 10 studies did not utilize any selection methods. Feature selection is recommended to improve model performance by eliminating irrelevant features, as suggested in (18, 88). However, studies like (22) suggest that powerful classification methods might handle high-dimensional datasets without requiring selection.

Both traditional and modern classification techniques were widely used, either individually or in combination. SVM emerged as the most popular traditional supervised method, aligning with findings in (20) for BC recurrence prediction. Additionally, studies like (22, 91) listed SVM among common BC prediction algorithms. Its popularity can be attributed to its generalization capabilities, excellent classification performance, and ability to handle high-dimensional data as discussed in (92).

Bagging algorithms, DNNs, and XGBoost were frequently used among modern methods. While (88) identified CNNs as the leading model for both binary and multiclass classification, similar to (87), the second most common algorithm reported in (22) was ANN. While the potential utility of advanced optimization techniques, including metaheuristic algorithms (93) game theory (94), and quantum computing (95), warrants investigation, none of the reviewed studies employed these approaches.

Traditional ML techniques such as DT, SVM, and LR are often more interpretable than DL models. This interpretability is vital in clinical environments where comprehending the decision-making process is essential for trust and validation. Traditional ML methods are also well-suited for smaller datasets, which are common in medical research due to the limited availability of patient data. Techniques like RF and SVM possess well-established theoretical foundations and are widely utilized, making them reliable choices for numerous applications.

However, traditional ML methods often necessitate extensive manual feature engineering, which can be labor-intensive and may not capture all pertinent patterns within the data. These methods may encounter difficulties with very large datasets or high-dimensional data, limiting their applicability in certain contemporary medical applications. While effective, traditional ML approaches may not achieve the same level of accuracy as DL methods, particularly in complex predictive tasks.

Deep learning methods, particularly CNNs and DNNs, have demonstrated superior performance in terms of accuracy for breast cancer survival prediction. DL models can autonomously extract relevant features from raw data, thereby reducing the necessity for manual feature engineering. This capability is particularly advantageous when dealing with complex data such as medical images. DL methods excel in handling large datasets and intricate data structures, rendering them suitable for applications involving high-dimensional data.

Nevertheless, DL models are often characterized as “black boxes” due to their lack of interpretability, which can pose a significant drawback in clinical settings where understanding the rationale behind predictions is crucial. DL methods typically require substantial amounts of labeled data for effective training, which can be a limitation in medical research where annotated data is scarce. Furthermore, training DL models is computationally intensive and demands considerable resources, which may not be accessible in all research contexts.

Hybrid models that integrate traditional ML and DL techniques leverage the strengths of both approaches. For example, they can employ DL for feature extraction and traditional ML for classification, resulting in enhanced performance and interpretability. These models can adapt to various types of data and often exhibit greater robustness in handling diverse clinical scenarios. However, hybrid models can be complex to design and implement, requiring expertise in both traditional ML and DL techniques. The integration of different model types can introduce challenges, particularly in ensuring that the strengths of each component are effectively harnessed.

The analysis highlights that although traditional ML and DL methodologies each have unique advantages and limitations, hybrid models offer a promising solution by integrating the strengths of both approaches. However, these models’ complexity and substantial resource demands are critical factors that must be considered. Future research should prioritize enhancing the interpretability and efficiency of DL models, alongside developing robust hybrid models that can be more effectively incorporated into clinical settings. By overcoming these challenges, the potential of artificial intelligence to enhance breast cancer survival prediction can be fully harnessed, leading to improved patient outcomes and more personalized treatment strategies.

Accuracy, due to its frequent use, was the primary metric for meta-analysis and performance comparison. Internal validation techniques were dominant, and employed in over 80% of the studies, with K-fold cross-validation being the most common approach. These findings align with other studies like (19, 20, 22). Notably, studies employing both internal and external validation achieved the highest mean validation accuracy.

Graphs or schematics were the most prevalent methods for visualizing BC survival prediction models (34.38%). Formulas, and their combination with graphs, were used in about 31% of the reviewed studies. This aligns with the findings of (22) who reported a combined formula and graph approach as a widely used presentation model.

The recent proliferation of AI techniques for classification tasks has undeniably led to an upsurge in publications exploring their implementation outcomes. Notably, while advanced analysis techniques are readily available, access to large, curated datasets reflecting real-world clinical data appears limited among researchers employing approaches discussed in this review. Among the momentous issues documented in our study, the appropriateness of dated datasets for the training and testing of traditional, modern, and hybrid ML algorithms was questionable. So that, replacing this data with new ones or mixing them with up-to-date data may alter outcomes. This issue should be considered and examined in forthcoming studies. Our meta-analysis suggests that recent hybrid approaches utilizing unsupervised modern algorithms, particularly when applied to image data, show promising potential. However, the heterogeneity observed in study designs across the included research limited the ability to definitively identify a single superior method. Future research with more homogenous datasets is warranted to validate these findings and further explore the capabilities of these emerging techniques in breast cancer survival prediction.

There is a discernible shift towards hybrid models that synergistically integrate traditional ML and DL techniques. These hybrid frameworks capitalize on the respective advantages of each approach, such as employing DL for sophisticated feature extraction and utilizing conventional ML for classification tasks, thereby enhancing both performance and interpretability. In particular, DL architectures, notably CNNs, are increasingly utilized for the automated extraction of features from intricate data types, such as medical imaging, which significantly diminishes the necessity for manual feature engineering. Combining diverse data modalities, including clinical, genomic, and imaging data, is becoming prevalent, offering a holistic understanding of breast cancer and augmenting predictive accuracy. There is a pronounced emphasis on the development of explainable AI models to mitigate the “black box” nature inherent in DL. Techniques such as SHAP and LIME are being investigated to enhance model interpretability. Furthermore, the adoption of transfer learning, wherein pre-trained models are fine-tuned on specific datasets, is gaining momentum. This strategy can substantially reduce the requisite data and computational resources for training.

Subsequent research endeavors should prioritize rigorous external validation to ascertain the robustness and generalizability of predictive models across heterogeneous populations. Conducting extensive prospective studies to validate the clinical efficacy of AI algorithms is imperative, as these investigations will facilitate the assessment of the real-world applicability of such models. Formulating strategies for the seamless integration of AI models into clinical workflows is crucial, encompassing the development of user-friendly interfaces and ensuring models function effectively in real-time clinical environments. Establishing ethical guidelines and regulatory frameworks is vital to guarantee the responsible deployment of AI in healthcare, addressing concerns related to data privacy, algorithmic bias, and transparency. Future research should also concentrate on devising cost-effective AI solutions that can be widely adopted, particularly in resource-constrained settings.

Implementing data augmentation techniques and fostering data sharing among research institutions can ameliorate the challenge of limited data availability. The development and incorporation of explainable AI techniques can enhance the interpretability of DL models, rendering them more acceptable in clinical practice. Leveraging cloud computing and federated learning can alleviate computational resource constraints by distributing the training process across multiple nodes. Emphasizing external validation using diverse and independent datasets can bolster the generalizability and robustness of AI models.

This study has some limitations. First, the search strategy was restricted to English articles, potentially excluding relevant non-English publications. Second, limited access to some full texts prevented their review.

This systematic review and meta-analysis provided valuable insights into the application of AI in BC survival prediction research. The findings highlighted the prevalence of traditional and modern ML methods, the importance of data quality and quantity, and the potential benefits of longer prediction horizons and feature selection techniques. Additionally, the review identified areas for improvement, such as the underutilization of data augmentation and the need for more robust validation strategies. Future research should address these limitations and strive to develop more generalizable and accurate ML models for BC survival prediction.

## 5 Conclusion

This systematic review and meta-analysis comprehensively analyzed the application of ML in BC survival prediction research. Our findings revealed the extensive use of diverse ML algorithms, achieving promising results. However, a critical limitation identified across studies was the predominant reliance on internal validation for performance evaluation. This restricts generalizability – a crucial factor for real-world clinical implementation. Future research should prioritize external validation on independent datasets and rigorous model benchmarking to enhance the applicability of ML models in BC survival prediction.

Furthermore, the analysis identified shortcomings in data utilization. Inappropriate datasets and a lack of essential pre-processing techniques, such as feature selection and data augmentation, were frequently observed. Addressing these issues through the appropriate selection and application of datasets and pre-processing methods in future studies is crucial for improving model performance and generalizability.

In conclusion, AI has emerged as a significant tool for BC survival prediction. However, further exploration and research are essential to fully understand the true impact and effectiveness of these methods. This study provides valuable insights into current research trends and methodological considerations. Researchers can leverage these findings to guide the design of future BC survival prediction projects and address the identified shortcomings of previous studies, ultimately propelling advancements in this domain.

## Data Availability

The original contributions presented in the study are included in the article/**Supplementary Material**. Further inquiries can be directed to the corresponding authors.
