# Supplementary material for: Artificial intelligence in breast cancer survival prediction: a comprehensive systematic review and meta-analysis
Source: Front Oncol. 2025 Jan 7;14:1420328. doi: 10.3389/fonc.2024.1420328 (PMC11747035; doi:10.3389/fonc.2024.1420328)
Supplement: Supplementary file 1 [file Table1.docx]

Appendix **A**

| **Search Strategy** | | |
| --- | --- | --- |
| **Database** | **Search Query** | |
| **PubMed** | #1 | (breast[Title]) |
|  | #2 | (cancer[Title]) OR (tumor*[Title]) OR (tumour*[Title]) OR (oncology[Title]) OR (neoplasm*[Title]) OR (malignanc*[Title]) OR (carcinoma*[Title]) |
|  | #3 | (surviv*[Title]) |
|  | #4 | (prognos*[Title]) OR (predict*[Title]) OR (decid*[Title]) OR (decision*[Title]) OR (detect*[Title]) OR (classif*[Title]) OR (classification*[Title]) |
|  | #5 | ("Linear Discriminant Analysis"[Title]) OR ("K nearest neighbor"[Title]) OR ("K nearest neighbors"[Title]) OR ("K nearest neighbour"[Title]) OR ("K nearest neighbours"[Title]) OR ("K means"[Title]) OR ("Decision Tree"[Title]) OR ("Decision Trees"[Title]) OR ("Random Forest"[Title]) OR ("Random Forests"[Title]) OR ("Naive Bayes"[Title]) OR ("Naive Bayesian"[Title]) OR ("Support Vector Machine"[Title]) OR ("Support Vector Machines"[Title]) OR ("Deep Learning"[Title]) OR ("Neural Network"[Title]) OR ("AdaBoost"[Title]) OR ("XGBoost"[Title]) OR ("Gradient Boosting"[Title]) OR ("Algorithm"[Title]) OR ("Machine Learning"[Title]) OR ("Supervised Learning"[Title]) OR ("Unsupervised Learning"[Title]) OR ("Supervised Machine Learning"[Title]) OR ("Unsupervised Machine Learning"[Title]) OR ("generative adversarial network"[Title]) OR ("artificial intelligence"[Title]) OR ("transfer learning"[Title]) |
|  | #6 | #1 AND #2 AND #3 AND #4 AND #5 AND 2016 OR 2017 OR 2018 OR 2019 OR 2020 OR 2021 OR 2022 OR 2023 (Publication Years) AND Article AND English (Languages) |
| **Web of Science** | #1 | TI=(((breast))) |
|  | #2 | TI=(((cancer) OR (tumor*) OR (tumour*) OR (oncology) OR (neoplasm*) OR (malignanc*) OR (carcinoma*))) |
|  | #3 | TI=(((surviv*))) |
|  | #4 | TI=(((anali*) OR (prognos*) OR (predict*) OR (decid*) OR (decision*) OR (detect*) OR (classif*) OR (classification*))) |
|  | #5 | TI=((("Linear Discriminant Analysis") OR ("K nearest neighbor") OR ("K nearest neighbors") OR ("K nearest neighbour") OR ("K nearest neighbours") OR ("K means") OR ("Decision Tree") OR ("Decision Trees") OR ("Random Forest") OR ("Random Forests") OR ("Naive Bayes") OR ("Naive Bayesian") OR ("Support Vector Machine") OR ("Support Vector Machines") OR ("Deep Learning") OR ("Neural Network") OR ("AdaBoost") OR ("XGBoost") OR ("Gradient Boosting") OR ("Algorithm") OR ("Machine Learning") OR ("Supervised Learning") OR ("Unsupervised Learning") OR ("Supervised Machine Learning") OR ("Unsupervised Machine Learning") OR ("generative adversarial network") OR ("artificial intelligence") OR ("transfer learning"))) |
|  | #6 | #1 AND #2 AND #3 AND #4 AND #5 AND 2016 OR 2017 OR 2018 OR 2019 OR 2020 OR 2021 OR 2022 OR 2023 (Publication Years) AND Article AND English (Languages) |
| **Scopus** | #1 | (TITLE ((breast))) |
|  | #2 | (TITLE ((cancer) OR (tumor*) OR (tumour*) OR (oncology) OR (neoplasm*) OR (malignanc*) OR (carcinoma*))) |
|  | #3 | (TITLE ((surviv*))) |
|  | #4 | (TITLE ((anali*) OR (prognos*) OR (predict*) OR (decid*) OR (decision*) OR (detect*) OR (classif*) OR (classification*))) |
|  | #5 | (TITLE (("Linear Discriminant Analysis") OR ("K nearest neighbor") OR ("K nearest neighbors") OR ("K nearest neighbour") OR ("K nearest neighbours") OR ("K means") OR ("Decision Tree") OR ("Decision Trees") OR ("Random Forest") OR ("Random Forests") OR ("Naive Bayes") OR ("Naive Bayesian") OR ("Support Vector Machine") OR ("Support Vector Machines") OR ("Deep Learning") OR ("Neural Network") OR ("AdaBoost") OR ("XGBoost") OR ("Gradient Boosting") OR ("Algorithm") OR ("Machine Learning") OR ("Supervised Learning") OR ("Unsupervised Learning") OR ("Supervised Machine Learning") OR ("Unsupervised Machine Learning") OR ("generative adversarial network") OR ("artificial intelligence") OR ("transfer learning"))) |
|  | #6 | #1 AND #2 AND #3 AND #4 AND #5 AND PUBYEAR > 2015 AND ((LIMIT-TO(DOCTYPE, "ar")) AND (LIMIT-TO(LANGUAGE, "English")) |
