# Supplementary material for: Artificial intelligence in breast cancer survival prediction: a comprehensive systematic review and meta-analysis
Source: Front Oncol. 2025 Jan 7;14:1420328. doi: 10.3389/fonc.2024.1420328 (PMC11747035; doi:10.3389/fonc.2024.1420328)
Supplement: Supplementary file 2 [file Table2.docx]

Appendix **B**

| Study | Unmet need | Feature engineering | Platforms | Hyper-parameters | Methods for over-fitting | Stability of results | External data validation | Predictors explanation | Suggested clinical use | Quality assessment score |
| --- | --- | --- | --- | --- | --- | --- | --- | --- | --- | --- |
| H. Li, et al. [1] | 1 | 1 | 1 | 1 | 1 | 1 | 1 | 1 | 1 | 9 |
| N. Arya, et al. [2] | 1 | 1 | 1 | 1 | 1 | 1 | 1 | 1 | 1 | 9 |
| S. Gupta, et al. [3] | 1 | 1 | 1 | 1 | 1 | 1 | 0 | 1 | 1 | 8 |
| W. Jang, et al. [4] | 1 | 1 | 1 | 1 | 1 | 1 | 0 | 1 | 1 | 8 |
| N. A. Othman, et al. [5] | 1 | 1 | 1 | 1 | 1 | 1 | 0 | 1 | 1 | 8 |
| C. C. Chang, et al. [6] | 1 | 1 | 1 | 1 | 1 | 1 | 0 | 1 | 1 | 8 |
| R. S. A. Maabreh, et al. [7] | 1 | 1 | 1 | 1 | 1 | 1 | 0 | 1 | 1 | 8 |
| E. Y. Kalafi, et al. [8] | 1 | 1 | 1 | 1 | 1 | 1 | 0 | 1 | 1 | 8 |
| H. Dammu, et al. [9] | 1 | 1 | 1 | 1 | 1 | 1 | 0 | 1 | 1 | 8 |
| A. Moncada-torres, et al. [10] | 1 | 1 | 1 | 1 | 1 | 1 | 0 | 1 | 1 | 8 |
| Q. T. N. Nguyen, et al. [11] | 1 | 1 | 1 | 0 | 1 | 1 | 1 | 1 | 1 | 8 |
| M. Zhao, et al. [12] | 1 | 1 | 1 | 1 | 1 | 1 | 0 | 1 | 1 | 8 |
| S. A. El Rahman [13] | 1 | 1 | 1 | 1 | 1 | 1 | 0 | 1 | 1 | 8 |
| T. R. Mahesh, et al. [14] | 1 | 1 | 1 | 1 | 0 | 1 | 1 | 1 | 1 | 8 |
| M. Salehi, et al. [15] | 1 | 1 | 1 | 1 | 1 | 1 | 0 | 1 | 1 | 8 |
| S. Z. Shahraki, et al. [16] | 1 | 1 | 1 | 1 | 1 | 1 | 0 | 1 | 1 | 8 |
| I. Mihaylov, et al. [17] | 1 | 1 | 1 | 1 | 1 | 1 | 0 | 0 | 1 | 7 |
| R. Albusayli, et al. [18] | 1 | 0 | 1 | 0 | 1 | 1 | 1 | 1 | 1 | 7 |
| E. Mustafa, et al. [19] | 1 | 1 | 1 | 1 | 1 | 1 | 0 | 0 | 1 | 7 |
| M. Montazeri, et al. [20] | 1 | 0 | 1 | 1 | 1 | 1 | 0 | 1 | 1 | 7 |
| L. Tapak, et al. [21] | 1 | 0 | 1 | 1 | 1 | 1 | 0 | 1 | 1 | 7 |
| Kim, j. Y, et al. [22] | 1 | 1 | 1 | 1 | 0 | 1 | 0 | 1 | 1 | 7 |
| S. Momenyan, et al. [23] | 1 | 0 | 1 | 1 | 1 | 1 | 0 | 1 | 1 | 7 |
| S. J. Lou, et al. [24] | 1 | 1 | 1 | 1 | 0 |  | 0 | 1 | 1 | 6 |
| K. Y. Huang, et al. [25] | 1 | 0 | 1 | 0 | 1 | 1 | 0 | 1 | 1 | 6 |
| V. R. Mudunuru, et al. [26] | 1 | 1 | 1 | 1 | 0 | 1 | 0 | 0 | 1 | 6 |
| H. Lotfnezhad afshar, et al. [27] | 1 | 1 | 1 | 1 | 0 | 0 | 0 | 1 | 1 | 6 |
| M. D. Ganggayah, et al. [28] | 1 | 1 | 1 | 1 | 0 | 0 | 0 | 1 | 1 | 6 |
| A. Tahmassebi, et al. [29] | 1 | 1 | 0 | 0 | 1 | 1 | 0 | 1 | 1 | 6 |
| P. Liu, et al. [30] | 1 | 1 | 1 | 1 | 0 |  | 0 | 1 | 1 | 6 |
| M. Takada, et al. [31] | 1 | 0 | 1 | 1 | 1 | 0 | 0 | 1 | 1 | 6 |
| L. C. Ji, et al. [32] | 1 | 0 | 1 | 0 | 0 | 1 | 1 | 0 | 1 | 5 |
|  |  |  |  |  |  |  |  |  |  |  |

[1] H. Li *et al.*, “Development and Validation of a New Multiparametric Random Survival Forest Predictive Model for Breast Cancer Recurrence with a Potential Benefit to Individual Outcomes,” *Cancer Management and Research,* vol. 14, pp. 909-923, 2022.

[2] N. Arya, and S. Saha, “Multi-modal advanced deep learning architectures for breast cancer survival prediction,” *Knowledge-Based Systems,* vol. 221, Jun, 2021.

[3] S. Gupta, and M. K. Gupta, “A Comparative Analysis of Deep Learning Approaches for Predicting Breast Cancer Survivability,” *Archives of Computational Methods in Engineering,* vol. 29, no. 5, pp. 2959-2975, Aug, 2022.

[4] W. Jang *et al.*, “Artificial intelligence for predicting five-year survival in stage IV metastatic breast cancer patients: A focus on sarcopenia and other host factors,” *Frontiers in Physiology,* vol. 13, Sep, 2022.

[5] N. A. Othman, M. A. Abdel-Fattah, and A. T. Ali, “A Hybrid Deep Learning Framework with Decision-Level Fusion for Breast Cancer Survival Prediction,” *Big Data and Cognitive Computing,* vol. 7, no. 1, Mar, 2023.

[6] C. C. Chang, and S. H. Chen, “Developing a Novel Machine Learning-Based Classification Scheme for Predicting SPCs in Breast Cancer Survivors,” *Frontiers in Genetics,* vol. 10, Sep, 2019.

[7] R. S. A. Maabreh, M. B. Alazzam, and A. S. AlGhamdi, “Machine Learning Algorithms for Prediction of Survival Curves in Breast Cancer Patients,” *Applied Bionics and Biomechanics,* vol. 2021, Nov, 2021.

[8] E. Y. Kalafi *et al.*, “Machine Learning and Deep Learning Approaches in Breast Cancer Survival Prediction Using Clinical Data,” *Folia Biologica,* vol. 65, no. 5-6, pp. 212-220, 2019.

[9] H. Dammu, T. M. Ren, and T. Q. Duong, “Deep learning prediction of pathological complete response, residual cancer burden, and progression-free survival in breast cancer patients,” *Plos One,* vol. 18, no. 1, Jan, 2023.

[10] A. Moncada-Torres *et al.*, “Explainable machine learning can outperform Cox regression predictions and provide insights in breast cancer survival,” *Scientific Reports,* vol. 11, no. 1, Mar, 2021.

[11] Q. T. N. Nguyen *et al.*, “Machine learning approaches for predicting 5-year breast cancer survival: A multicenter study,” *Cancer Sci*, Jul 25, 2023.

[12] M. Zhao *et al.*, “Machine Learning With K-Means Dimensional Reduction for Predicting Survival Outcomes in Patients With Breast Cancer,” *Cancer Informatics,* vol. 17, Nov, 2018.

[13] S. A. El Rahman, “Predicting breast cancer survivability based on machine learning and features selection algorithms: a comparative study,” *Journal of Ambient Intelligence and Humanized Computing,* vol. 12, no. 8, pp. 8585-8623, Aug, 2021.

[14] T. R. Mahesh *et al.*, “Performance Analysis of XGBoost Ensemble Methods for Survivability with the Classification of Breast Cancer,” *Journal of Sensors,* vol. 2022, Sep, 2022.

[15] M. Salehi *et al.*, “A One-Dimensional Probabilistic Convolutional Neural Network for Prediction of Breast Cancer Survivability,” *Computer Journal,* vol. 65, no. 10, pp. 2641-2653, Oct, 2022.

[16] S. Z. Shahraki *et al.*, “Time-related survival prediction in molecular subtypes of breast cancer using time-to-event deep-learning-based models,” *Frontiers in Oncology,* vol. 13, Jun, 2023.

[17] I. Mihaylov, M. Nisheva, and D. Vassilev, “Application of machine learning models for survival prognosis in breast cancer studies,” *Information (Switzerland),* vol. 10, no. 3, 2019.

[18] R. Albusayli *et al.*, “Artificial intelligence-based digital scores of stromal tumour-infiltrating lymphocytes and tumour-associated stroma predict disease-specific survival in triple-negative breast cancer,” *Journal of Pathology,* vol. 260, no. 1, pp. 32-42, May, 2023.

[19] E. Mustafa *et al.*, “An Ensembled Framework for Human Breast Cancer Survivability Prediction Using Deep Learning,” *Diagnostics,* vol. 13, no. 10, May, 2023.

[20] M. Montazeri *et al.*, “Machine learning models in breast cancer survival prediction,” *Technology and Health Care,* vol. 24, no. 1, pp. 31-42, 2016.

[21] L. Tapak *et al.*, “Prediction of survival and metastasis in breast cancer patients using machine learning classifiers,” *Clinical Epidemiology and Global Health,* vol. 7, no. 3, pp. 293-299, Sep, 2019.

[22] J.-Y. Kim *et al.*, “Deep learning-based prediction model for breast cancer recurrence using adjuvant breast cancer cohort in tertiary cancer center registry,” *Frontiers in oncology,* vol. 11, pp. 596364, 2021.

[23] S. Momenyan *et al.*, “Survival Prediction of Patients with Breast Cancer: Comparisons of Decision Tree and Logistic Regression Analysis,” *International Journal of Cancer Management,* vol. 11, no. 7, Jul, 2018.

[24] S. J. Lou *et al.*, “Breast Cancer Surgery 10-Year Survival Prediction by Machine Learning: A Large Prospective Cohort Study,” *Biology-Basel,* vol. 11, no. 1, Jan, 2022.

[25] K. Y. Huang *et al.*, “The impact of chemotherapy and survival prediction by machine learning in early Elderly Triple Negative Breast Cancer (eTNBC): a population based study from the SEER database,” *Bmc Geriatrics,* vol. 22, no. 1, Apr, 2022.

[26] V. R. Mudunuru, and L. A. Skrzypek, “A Comparison of Artificial Neural Network and Decision Trees with Logistic Regression as Classification Models for Breast Cancer Survival,” *International Journal of Mathematical Engineering and Management Sciences,* vol. 5, no. 6, pp. 1170-1190, Dec, 2020.

[27] H. Lotfnezhad Afshar *et al.*, “Prediction of Breast Cancer Survival by Machine Learning Methods: An Application of Multiple Imputation,” *Iranian Journal of Public Health,* vol. 50, no. 3, pp. 598-605, Mar, 2021.

[28] M. D. Ganggayah *et al.*, “Predicting factors for survival of breast cancer patients using machine learning techniques,” *Bmc Medical Informatics and Decision Making,* vol. 19, Mar, 2019.

[29] A. Tahmassebi *et al.*, “Impact of Machine Learning With Multiparametric Magnetic Resonance Imaging of the Breast for Early Prediction of Response to Neoadjuvant Chemotherapy and Survival Outcomes in Breast Cancer Patients,” *Investigative Radiology,* vol. 54, no. 2, pp. 110-117, Feb, 2019.

[30] P. Liu *et al.*, “Optimizing Survival Analysis of XGBoost for Ties to Predict Disease Progression of Breast Cancer,” *Ieee Transactions on Biomedical Engineering,* vol. 68, no. 1, pp. 148-160, Jan, 2021.

[31] M. Takada *et al.*, “Prediction of postoperative disease-free survival and brain metastasis for HER2-positive breast cancer patients treated with neoadjuvant chemotherapy plus trastuzumab using a machine learning algorithm,” *Breast Cancer Research and Treatment,* vol. 172, no. 3, pp. 611-618, Dec, 2018.

[32] L. C. Ji *et al.*, “Osteoporosis, fracture and survival: Application of machine learning in breast cancer prediction models,” *Frontiers in Oncology,* vol. 12, Aug, 2022.
